# Impact of SNPs on methylation readouts by Illumina Infinium HumanMethylation450 BeadChip Array: implications for comparative population studies

**DOI:** 10.1186/s12864-015-2202-0

**Published:** 2015-11-25

**Authors:** Patrycja Daca-Roszak, Aleksandra Pfeifer, Jadwiga Żebracka-Gala, Dagmara Rusinek, Aleksandra Szybińska, Barbara Jarząb, Michał Witt, Ewa Ziętkiewicz

**Affiliations:** Institute of Human Genetics, Polish Academy of Sciences, Poznan, Poland; Department of Nuclear Medicine and Endocrine Oncology, Maria Skłodowska-Curie Memorial Cancer Center and Institute of Oncology, Gliwice Branch, Gliwice, Poland; International Institute of Molecular and Cell Biology, Warsaw, Poland

**Keywords:** Human DNA methylation, Genomic SNPs, CpG sites, β-values distribution, Allele frequency, Illumina platform, Infinium probes

## Abstract

**Background:**

Infinium HumanMethylation 450 BeadChip Arrays by Illumina (Illumina HM450K) are among the most popular CpG microarray platforms widely used in biological and medical research. Several recent studies highlighted the potentially confounding impact of the genomic variation on the results of methylation studies performed using Illumina’s Infinium methylation probes. However, the complexity of SNPs impact on the methylation level measurements (β values) has not been comprehensively described.

**Results:**

In our comparative study of European and Asian populations performed using Illumina HM450K, we found that the majority of Infinium probes, which differentiated two examined groups, had SNPs in their target sequence. Characteristic tri-modal or bi-modal patterns of β values distribution among individual samples were observed for CpGs with SNPs in the first and second position, respectively. To better understand how SNPs affect methylation readouts, we investigated their impact in the context of SNP position and type, and of the Illumina probe type (Infinium I or II).

**Conclusions:**

Our study clearly demonstrates that SNP variation existing in the genome, if not accounted for, may lead to false interpretation of the methylation signal differences suggested by some of the Illumina Infinium probes. In addition, it provides important practical clues for discriminating between differences due to the methylation status and to the genomic polymorphisms, based on the inspection of methylation readouts in individual samples. This approach is of special importance when Illumina Infinium assay is used for any comparative population studies, whether related to cancer, disease, ethnicity where SNP frequencies differentiate the studied groups.

## Background

Human DNA methylation studies have become an important part of many biological and medical research areas [1, 2]. A number of methods for analyzing genome-wide methylation profiles have been developed and validated [3–9]. Illumina Infinium HumanMethylation 450 BeadChip Array (HM450K array) is one of the most popular CpG microarray platforms used to quantify genome-wide methylation status following bisulfate treatment of the analyzed DNA.

The number of methylation studies performed with the use of Illumina HM450K array have been published in various research fields, including studies on cancer, disease, ethnicity, aging, and gender, and it still grows exponentially [1, 10–15]. Great popularity of the Illumina platform stems from its ability to test methylation at the genome-wide scale (over 480 000 predefined CpG sites) combined with the relatively low cost of the assay.

Recently, some technical limitations of the Infinium platform have been recognized, leading to the recommendation for a more careful interpretation of DNA methylation results from the HM450K array [12, 16–19]. The limitations reflect suboptimal probe design, e.g. probes hybridizing to multiple map addresses, probes located within repetitive sequences, or probes targeting genomic sequence containing SNPs. All these factors affect determination of β or M values (measures of the methylation level); if not accounted for, they may lead to false interpretation of Illumina microarray results. Recently, the list of Illumina HM450K probes likely to provide a “noisy” signal (with the recommendation for being discarded from methylation studies) was published [19]. Among a total of 485,512 Illumina probes, 70,118 were affected by SNPs or INDELS at interrogated CpG sites, including 12,746 Infinium I and 57,372 Infinium II probes. While very helpful, the study by Naeem et al. [19] does not characterize the impact of specific categories of potentially confounding factors on the interpretation of Illumina HM450K results in the context of population studies.

The presence of SNPs at the CpG loci targeted by Illumina Infinium probes is one of the most important confounding variables, especially when methylation levels are compared among individuals from genetically non-homogeneous populations. Our interest in comparative population genetics prompted us to characterize the theoretical and practical impact of SNPs on the methylation readouts and on the interpretation of population data. Using exemplary results obtained with the use of Infinium probes in male B-lymphocyte lines from two human continental populations: Caucasian (European) and East Asian (Chinese), we illustrate how SNPs at the interrogated CpG loci affect the resulting methylation calls. Our study provides important practical clues for using HM450K array in comparative population studies.

## Results

### Selection of differentiating probes

Methylation microarray analysis of 36 European and East Asian cell lines revealed a total of 34,024 sites differentiating two populations) at the significance level *p* < 0.05. Of those, 120 CpG sites were characterized by q < 0.05, and arbitrarily set |M_av__diff| > 0.3. These sites included 33 CpGs targeted by Infinium probes type I and 87 by probes type II, and were used to demonstrate the impact of genomic SNPs on the interpretation of methylation readout from HM450K array. A subset of 96 CpG sites (23 and 73 probes type I and II, respectively), representing the highest inter-population differences (|M_av__diff| > 1; q < 0.05) was provisionally termed “population-differentiating” (pop-diff) CpGs. The cut-off at |M_av__diff| > 1 roughly corresponded to that of an absolute difference in β value > 0.2 [20], representing approximately a difference in DNA methylation levels of 20 %.

There was no global methylation difference between interrogated populations (*p* = 0.67).

### Genomic location of differentiating probes

We examined the 96 pop-diff probes (M_diff_ > 0.1; q < 0.05) for the presence of neighboring probes, located 500 bp up- or downstream from the investigated CpG. 53 of the 96 pop-diff probes had no other probes in the vicinity; the remaining 43 had from one to six neighbors. Altogether, there were 106 additional probes, 33 of which (31 %) differentiated two populations (M_diff_ 0.2–0.49); however, the majority of these were not included in the list of 96 pop-diff because they did not fulfill the requirement of q < 0.05.

Among 96 pop-diff probes, only two pairs (with no SNP in the interrogated CpG) mapped to the same chromosome region each. cg04287289 and cg26513180 were 8 bp apart on chromosome 16 (M_diff_ values 1.96 and 1.62 respectively); cg26367031 and cg00862290 were 226 bp apart on chromosome 3 (M_diff_ values 2.77 and 2.78 respectively). The remaining pop-diff probes, when localized on the same chromosome, were separated from one another by much larger distances (for 68 probes the distance was > 126 kb, for 5: between 1.8 and 74.8 kb).

### Probes targeting CpG sites with genomic SNPs

Among 96 pop-diff CpGs representing the highest inter-population differences between Europeans and Asians (|M_av__diff| > 1; q < 0.05), we frequently observed a high proportion of probes with a tri-modal or bi-modal distribution of the individual β values. This distribution resembled clustering expected for heterozygous and homozygous forms of SNP genotypes, also reported in earlier studies [16, 19].

Indeed, *in silico* analysis of the sequences in the UCSC Genome Browser database revealed that the majority (66/96, i.e. 69 %) of the pop-diff CpG sites contained common genomic SNPs (the minor allele frequency MAF > 0.01) with the allele frequencies strongly differentiating two analysed populations (MAF_diff > 0.1). Fst values calculated for each of these SNPs were in the range of 0.51–0.013.). The average genetic distance between European and East Asian populations based on these 66 SNPs was 0.26 (+ − 0.11), which is significantly more than the average Fst of 0.10–0.15 or less, reported for inter-continental comparisons of human populations (e.g. [21] and references therein).

In contrast, among 24 weakly differentiating CpG sites (M_av__diff < 1; q < 0.05), only 25 % had a SNP in the interrogated CpG. These observations suggested that the large proportion of population differences detected by the HM450K probes reflected genomic rather than epigenetic differences. None of these CpGs corresponded to the 65 control Illumina probes (rs) that assay highly-polymorphic single nucleotide polymorphisms (SNPs) and are intentionally included in the HM450K array to allow sample quality control and indicate the degree of technical variance between samples. All the probes targeting pop-diff CpGs with SNPs in the interrogated sequence belonged to the class flagged to be discarded from methylation analyses by Naeem et al. [19].

**Fig. 1:**
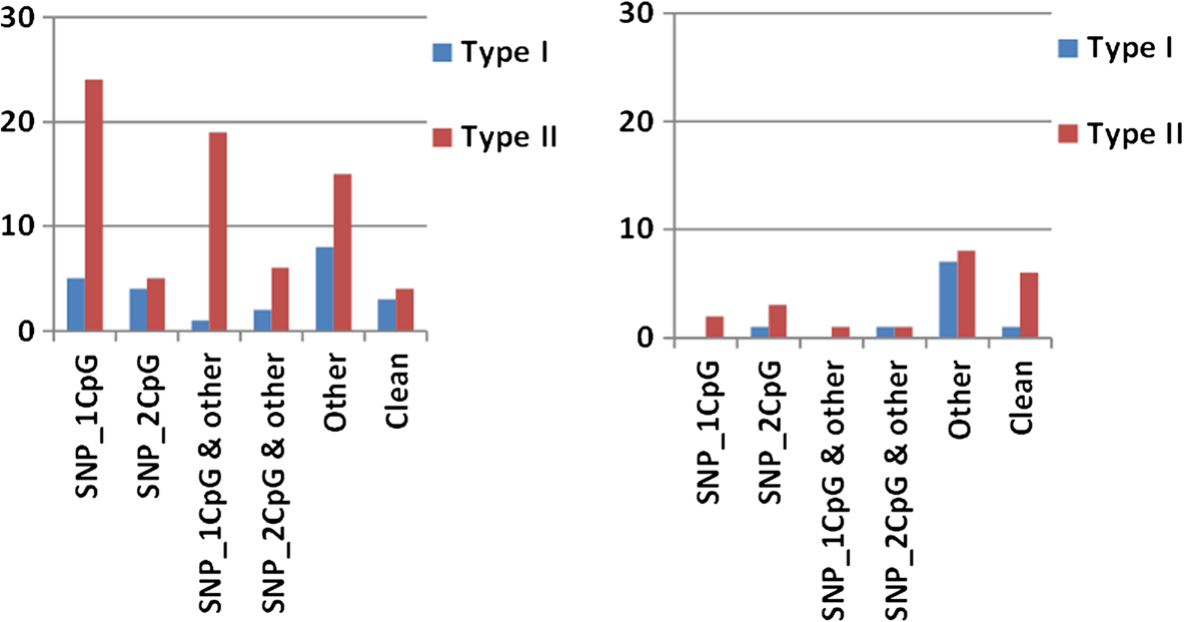
Occurrence of probes from the set of 120 characterized by FDR q < 0.05. **a**. Pop-diff probes (M_av__diff >1). **b**. Weakly differentiating probes (M_av__diff <1). Vertical axis — count of probes targeting CpG sites divided into categories listed along the horizontal axis: SNP_1CpG or SNP_2CpG — differentiating SNP in the first or second position of the interrogated CpG as the only confounding factor; SNP_(1,2) CpG & other — SNP in the interrogated CpG combined with other confounding factors; Other — probes with the confounding factors other than SNP in the interrogated CpG (SNPs in the probe sequence outside the interrogated CpG, indels, multi-map, repeats, mixed etc.); Clean — probes with no confounding factor, potentially the only probes indicating true methylation level differences

The proportion of pop-diff CpGs containing frequent SNPs was higher for type II (54/73; 74 %) than for type I Infinium probes (12/23; 52 %). When the position of a SNP within the interrogated CpG was considered, seven type I pop-diff probes had SNP in the first, and five—in the second CpG position; for type II pop-diff probes, 46 had SNP in the first, and eight—in the second CpG position. No significant differences between the number of probes with SNPs in the first and second CpG position were observed in a set of 24 weakly differentiating probes. This comparison clearly indicated that the population-differentiating capacity was mainly restricted to type II probes, with a frequent SNP in the first position of the interrogated CpG. This effect was seen both when a SNP in CpG was the only confounding factor, and when it was combined with other factors listed by Naeem et al. [19], like under-a-probe SNPs, probes localizing to the genomic repeats, or mapping at different genomic locations etc. (Fig. 1).

The excess of Infinium type II among the pop-diff probes with SNPs can be related to the fact that most of the CpGs interrogated by these probes are located in the genomic sea, outside of the presumably more evolutionary conserved CpG islands and shores (regions of ~2 kb adjacent to CpG islands, as defined by Illumina) interrogated by Infinium I probes.

### The impact of SNPs in the interrogated CpG sites on the pattern of β values distribution

In our study, the majority of pop-diff Infinium probes targeting CpG sites with common SNPs, yielded characteristic patterns of β values distribution among the examined cell lines: tri-modal, bi-modal, and cloud-like. In order to facilitate interpretation of such distributions, we examined potential factors influencing these patters. For individual probes, the pattern depended on: the SNP position in the interrogated CpG site; the probe type (Infinium I or II); and the methylation status of the wild type CpG allele at the examined locus. It has to be emphasized that in over 40 % of the pop-diff probes with a SNP in the targeted dinucleotide, other potentially confounding features were present; however, for most of them the presence of a SNP in the interrogated CpG appeared to exert the strongest effect upon methylation readouts. For the sake of clarity, examples presented below represent probes with SNPs in the interrogated CpG sites and with no other confounding factors. The examples are not intended to cover the whole range of variability in β values distribution. Instead, they are chosen to illustrate some of the mechanisms that influence the readouts, and possibly to help in the interpretation of some ambiguous results that may be encountered when using Illumina Infinium probes.

### Tri-modal distribution of β values

**Fig. 2:**
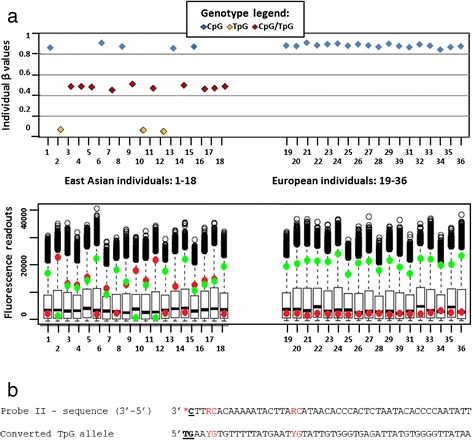
Example of a tri-modal pattern of β values distribution. **a**. *Upper panel*: Individual β values for 36 cell lines examined using the probe type II (cg23556238) interrogating CpG site with a SNP (C>T) in the first position. Vertical axis — individual β values; horizontal axis — individual cell lines. Samples 1–18 – East Asian population, samples 18–36 –European population. High, low, and intermediate β values are observed, respectively, in the cell lines with CpG (blue), TpG (yellow) and CpG/TpG (red) genotypes. The tri-modal distribution is that of beta values (along the Y axis). *Lower panel*: Box plot graph showing fluorescence readouts for the probe cg23556238. The normalized signals (values indicated on the vertical axis) are detected as a result of probe extension with differently labelled dideoxynucleotides: green and red, indicating methylated and unmethylated status, respectively. All lines with homozygous CpG genotypes display strong green and no red signal, indicating methylated status; lines with homozygous TpG genotypes display strong red and no green signal, mimicking unmethylated status; heterozygotes have equal intensity of both red and green fluorescence signals. Each boxplot represents interquartile range of the signals from all the probes for a given cell line. The bottom and top of the box represent the first and the third quartile, respectively, and the black line inside the box corresponds to the median. Black circles in the upper part of the graph are outlier values (>1.5 distance from the upper quartile). **b**. Sequence of the Infinium II probe (cg23556238), aligned with the genomic sequence. According to TpG genotype, dideoxy-A will be incorporated giving red fluorescence signal falsely indicating unmethylated status. “RC” in the probe sequence represents degenerated sites complementary to under-a-probe CpGs (“YG” in the BCD sequence indicates no assumption of the concordant methylation with the interrogated dinucleotide)

The most conspicuous and very frequent pattern observed among pop-diff CpGs in our study, was that of a tri-modal distribution of β values (see example in Fig. 2a). This pattern was typically seen when a C/T SNP was present in the first position of the dinucleotide interrogated by type II Infinium probes (see Fig. 2b). Individuals with the TpG genotype had low, CpG/TpG heterozygotes—intermediate, and CpG homozygotes—high β values. Therefore, the tri-modal distribution of β values reflected distribution of the SNP genotype frequencies in the examined populations.

While low β values observed for TpG alleles reflected the genomic status of the polymorphic site rather than the low level of methylation, high β values for CpG homozygotes indicated that CpG alleles were strongly methylated; if CpG alleles in polymorphic CpG sites were not methylated, both alleles would be indistinguishable after conversion, resulting in uniformly low β values due to the lack of the green (methylation-specific) fluorescence signal from the probes.

A similar tri-modal distribution was also observed when C in the first position of the interrogated CpG sites was substituted by A or G. In most CpG sites with a SNP in the first position, the concurrent presence of another confounding factor, like SNPs, indels or repeats under a probe, had only minor effect compared to the influence of SNP in the interrogated site and did not disturb the tri-modal distribution of β values (not shown).

### Bi-modal distribution of β values

**Fig. 3:**
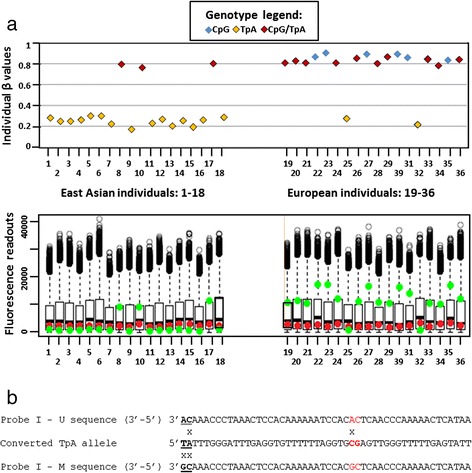
Example of a bi-modal pattern of β values distribution. **a**. *Upper panel*: Individual β values for 36 cell lines examined using the type I probe (cg16310958) interrogating CpG site with a SNP (G>A) in the second position. High β values are observed in cell lines with CpG (blue) and CpG/TpA (red) genotypes, low—in lines with CpA (TpA in converted DNA; yellow) genotypes. Similar bi-modal distributions were also observed when CpG sites with SNPs in the second position were interrogated by type II probes (not shown). *Lower panel*: Fluorescence readouts for the probe cg16310958. Signals are detected as a result of the extension of probes M (green) and U (red). All lines with homozygous CpG genotypes display strong green and no red signal, indicating methylated status. In heterozygotes the only readable signal is that from M probes. In lines with homozygous CpA (TpA in converted DNA) genotypes, fluorescence signals from both M and U probes are very low. **b**. Sequences of the cg16310958 probes M and U, aligned with the genomic sequence with the G>A substitution in the second position of the interrogated dinucleotide (TpA allele- bold and underlined). Additional CpG site under the probes (marked in red) appear to have the same methylation status as the CpG allele in the interrogated site. Neither U nor M probe would hybridize efficiently to the TpG allele

Another striking pattern, that of a bi-modal β distribution, was typically seen in CpGs with a G/A SNP at the second position (Fig. 3a- upper panel), interrogated by either type I or type II Infinium probes. In most cases, CpG homozygotes had high β values suggesting strong methylation of the interrogated dinucleotide. In CpA alleles, we intuitively expected β values to be very low, indicating non-methylated status of the interrogated TpA dinucleotide (CpA allele, being no longer “methylable”, is converted to TpA after bisulfite treatment). Surprisingly, β values in CpA homozygotes were raised, for some probes even up to the mid-level; at the same time, β values in heterozygotes did not differ from those obtained for CpG homozygotes. To explain this shift towards high β values, observed in all genotypes containing allele with a SNP in the second position of the interrogated CpG site, we looked closer at the possible factors, which could influence fluorescence readouts and the calculation of β values.

Inefficient hybridization of the probes to the converted TpA template resulted in the lack of probe extension, and thus very low residual fluorescence signals, both signalMeth and signalUnmeth (see data in Fig. 3a lower panel). This was true for both type I and type II Infinium probes. In type II probes, the lack of both red and green signals (indicating methylated and unmethylated status, respectively) resulted from the mismatch between the T in the interrogated dinucleotide (TpA in the converted DNA) and the probe’s sequence, always ending in C (see Tables 1 and 2). For Infinium type I, both M and U probes were doubly mismatched at their 3′ ends (Fig. 3b).

**Table 1:**
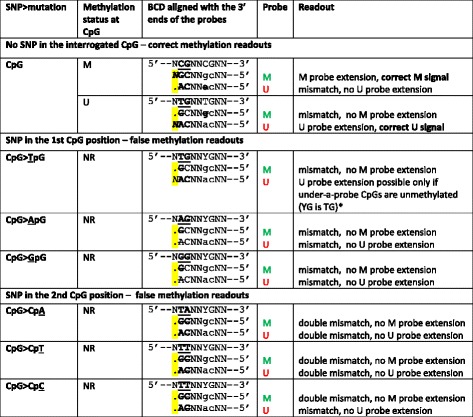
Predicted impact of SNPs in the interrogated CpG on methylation readout using Infinium I probes

*see also Fig. 4c.

Interrogated CpG sites are underlined and bolded. BCD — bisulfite converted DNA; NR — not relevant (no CpG in the genomic sequence); strikethrough — mismatch between the probe and the interrogated locus; highlighted italics — incorporated fluorescently labeled ddNTPs; highlighted dots adjacent to the 3′-most position of the probe — no probe extension; lower case “gc” and “ac” in the, respectively, M and U probe sequences should be complementary to CpGs and TpGs under a probe (methylation status concordant with that of the interrogated dicnucleotide); “YG” in the BCD sequence indicates that the methylation status of the under-a-probe CpGs cannot be predicted when the interrogated dinucleotide contains SNP

**Table 2:**
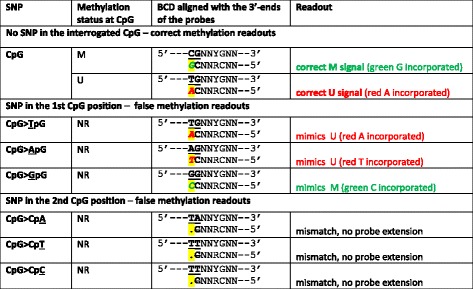
Predicted impact of SNPs in CpG on methylation readout using Infinium II probes

BCD - bisulfite converted DNA; NR – not relevant (no CpG in the genomic sequence); strikethrough – mismatch between the probe and the interrogated locus; italics – incorporated fluorescently labeled ddNTPs (C, G – green = methylation signal; A, T – red = unmethylation signal); dots adjacent to the 3′-most position of the probe indicate no probe extension; “RC” in the probe sequence represents degenerated sites complementary to under-a-probe CpGs (“YG” in the BCD sequence indicates no assumption of the concordant methylation with the interrogated dinucleotide)

In CpG/CpA (CpG/TpA after conversion) heterozygotes therefore, the only significant contribution to the fluorescence readout was from the probes indicating methylation (green signal), and β values did not differ from those obtained for methylated CpG homozygotes (see Fig. 3a – upper panel-right part). In CpA (TpA after conversion) homozygotes, with no contribution from the methylated CpG allele, both fluorescent signalMeth and signalUnmeth were very low compared to those in CpG homozygotes or in heterozygotes (see Fig. 3a- lower panel). These low signals, when entered in the equation β = signalMeth/(signalMeth + signalUnmeth), artificially inflated the respective β values.

### Distribution of β values for probes with abnormally low fluorescence signals

**Fig. 4:**
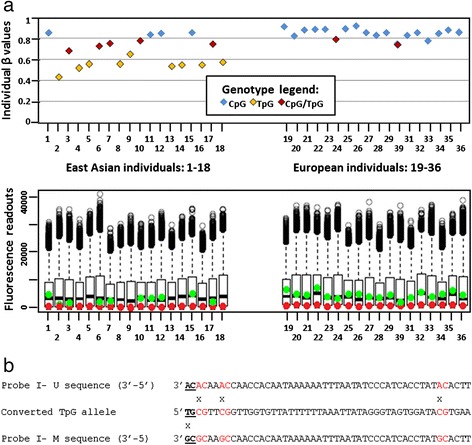
Example of the effect of abnormally low fluorescence readouts. **a**. *Upper panel*: Individual β values for 36 cell lines examined using the type I probe (cg10428733) interrogating CpG site with a SNP (C>T) in the first position. Only high and intermediate β values are observed, in cell lines with CpG (blue), TpG (yellow) and CpG/TpG (red) genotypes. *Lower panel*: Fluorescence readouts for the probe cg10428733. Signals are detected as a result of the extension of M (green) and U (red) probes. All lines with homozygous CpG genotypes display strong green and no red signal, indicating methylated status. In lines with homozygous TpG genotypes fluorescence signals from both probes are extremely low (only one colour is visible, due to the overlapping red and green signals). In heterozygotes the only readable signal comes from M probes. **b**. Sequences of the cg10428733 probes M and U, aligned with the genomic sequence (shown in opposed orientation) with the C>T substitution in the first position of the interrogated dinucleotide (bold and underlined). Three additional CpG sites under the probes (marked in red) appear to have the same methylation status as the CpG allele in the interrogated site. Neither U nor M probe would hybridize efficiently to the TpG allele: the M probe due to the mismatch at the interrogated TpG, and the U probe in addition, due to the mismatch with methylated under-a-probe CpGs, localized close to the 3′ end of the probe.

An artificial increase of β values (misleadingly suggesting methylation status), as described above, affected both type I and type II probes interrogating CpG site with a G/A SNP in the second position. A similar effect was observed when Infinium type I probes were used to interrogate CpG sites with a C/T SNP in the first position. Here, the mechanism of low fluorescence signals reflected the nature of Infinium type I probes, which assume concordant methylation of the neighbouring CpGs. In the example illustrated in Fig. 4a, high β values for CpG homozygotes indicated that under-a-probe CpGs were methylated. The presence of a TpG allele in the interrogated dinucleotide resulted in the mismatch of both U and M probes ( Fig. 4b); inefficiently hybridizing probes failed to extend, and very low fluorescence signals were observed (Fig. 4a; lower panel). In consequence, β values in all TpG-containing genotypes were artificially inflated, which resulted in a flattened pattern, with all β values clustering in the upper range (Fig. 4a- upper panel).

### A cloud-like distribution of β values

**Fig. 5:**
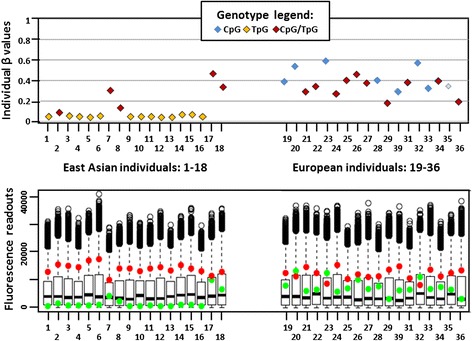
Examples of an ambiguous impact of SNP on the distribution of β values. *Upper panel*: Individual β values for 36 cell lines examined using the type II probe (cg14175932) interrogating CpG site with a SNP(C>T) at the first position. Low β values observed in most of the East Asian cell lines are concordant with the predominant presence of TpG alleles (yellow) in this population. The dispersed, intermediate β values observed in European population correspond to cell lines with CpG (blue) and CpG/TpG (red) genotypes; grey tags denote genotypes not confirmed experimentally. The varying β values for heterozygotes and CpG homozygotes indicate partial methylation status of the CpG alleles. *Lower panel*: Fluorescence readouts for the probe cg14175932 (Infinium II). Cell lines with homozygous TpG genotypes display strong red and no green signal, mimicking unmethylated status; heterozygotes have nearly-equal, medium-range intensity of both red and green fluorescence signals; all lines with homozygous CpG genotypes display similar green and red signal, indicating non-uniform methylation status

For a number of pop-diff probes, the impact of substitutions in the interrogated CpGs was ambiguous, with no clear correlation between the β values and the SNP genotypes. A typical characteristics of these patterns was a cloud-like pattern, with varying level of β values for CpG homozygotes, which indicated non-uniform level of CpG methylation. In these cases, β values for heterozygotes were variable, and homozygotes for non-CpG alleles usually had low β values (Fig. 5).

**Fig. 6:**
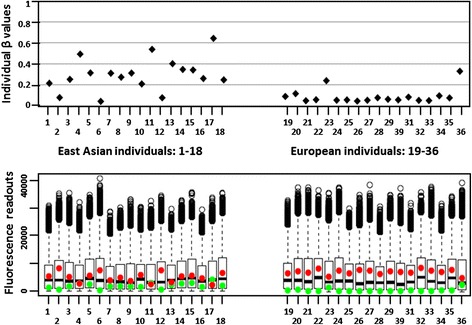
Example of β values distribution for one of the “clean” Infinium probes (with no SNP in the interrogated CpG site and no other confounding effect). *Upper panel*: Individual β values for the type I probe (cgx* — the probe’s name is not disclosed — validation study in progress; manuscript in preparation). *Upper panel*: Fluorescence readouts for the probe cgx. In the absence of SNPs, strong red and no green signal indicate unmethylated status, while similar, medium-range intensity of green and red signals indicates a partial methylation status

It has to be emphasized that similar cloud-like patterns of individual β values distribution were also observed when probes targeted “clean” CpGs (with no SNPs or other confounding factors). In these cases, inter-population differences in β_av_ values could be ascribed to real differences in the level of methylation between the studied groups (the need for further validation notwithstanding). In the example presented in Fig. 6, individual β values were very low in European samples, while in Asians, they were distributed between 0 and 60 % of the full methylation status. This probe is currently under validation by our research team, with the use of pyrosequencing (to be published).

## Discussion

### The predicted impact of SNPs on the methylation readout

The impact of SNPs on methylation readouts (β values) in the Illumina HM450K array, while predicted, has not been well characterized so far. Here we analyzed the theoretical, expected outcome in the context of different SNPs and probes, and compared it to the observed distribution of β values in the examined cell lines.

Among the factors that influence the impact of SNPs on the β values, the polymorphism’s location and type (in the interrogated dinucleotide or under a probe; affecting the first or the second position of the CpG) are the most obvious. Furthermore, because of the differences in the chemistry underlying signal detection when using Infinium I and II probes, SNP’s effect strongly depends on the type of Infinium probes (see Tables 1 and 2). Any SNP effect will be also influenced by the presence/absence of other factors, like indels, probe’s mapping to multiple locations, probes localized within genomic repeats, inconsistency with Whole Genome Bisulfite Sequencing (WGBS sequencing) (e.g. [19]). Here, for the sake of clarity, we only focused on the analysis of probes with SNPs, with no other confounding factors.

### SNPs under a probe

Among a variety of possible simple polymorphisms, the C>T or T>C substitutions, if present outside CpG dinucleotides, have no effect on the methylation readouts. This is due to the bisulfite treatment, which converts all Cs not involved in CpGs to Ts, such that the converted sequence behaves as if it was non-polymorphic (classified as “bisulfite-OK” by [19]). Any other substitutions located under a probe may affect the stability of probe’s hybridization and thus its extension efficiency; the effect of a mismatch would be stronger for SNPs located close to the 3′ end of the probe. In addition, if under-a-probe SNPs occur in CpG dinucleotides, their destabilizing effect would be stronger for the type I probe pairs (M and U, which assume concordant methylation status of the adjacent CpGs) and less obvious for the degenerated type II probes.

The effect of under-a-probe SNPs is not uniform and cannot be easily predicted from the probe sequence and sample genotype. This is reflected by the inconsistencies in the literature: some authors had suggested that SNPs within 10 bp of the interrogated CpG can affect probes [16], while others claimed that SNPs in the probe body may have little effect on the recorded methylation state [19].

### SNPs located in the interrogated CpGs

SNPs located in the interrogated CpGs have a potential to affect the Illumina HM450K array readouts in a more direct manner. Tables 1 and 2 summarize the predicted effects of SNP polymorphisms in the first and second position of the CpG site on the methylation readouts when interrogated by type II and I probes, respectively.

### Substitutions in the first position of the interrogated CpG site

For type I probes, the genomic C>T substitution in the interrogated CpG site renders it undistinguishable from the bisulfite-converted unmethylated dinucleotide (TpG). However, it has to be remembered that if the interrogated CpG was methylated, the C>T substitution would change the presumed concordance in the methylation status between the tested dinucleotide and under-a-probe CpGs. This would lead to an inefficient attachment of not only one but of both probes. The resulting low fluorescence readouts from both probes, compared to relatively high readouts from the same probes efficiently hybridizing with CpG alleles, would affect β values and return false estimates of the methylation level (see examples in the section “Distribution of β values for probes with abnormally low fluorescence signals”). The influence of the C>A or C>G substitutions in the interrogated CpG sites is difficult to predict; these substitutions would affect hybridization of the probe at its 3′ end, thus lowering the extension efficiency of both M and U probes.

For type II probes, the genomic C>T in the interrogated CpG site also mimics the unmethylated status of the examined dinucleotide in the bisulfite-converted DNA, resulting in the red fluorescence signal (incorporation of a complementary A). The genomic C>A substitution would give a similar signal (the red fluorescence signals from the incorporation of A or T are not distinguishable); the genomic C>G, in turn, would mimic the methylated status of the dinucleotide (the green fluorescence signals from the incorporation of C or G are also not distinguishable). Unlike in type I probes, the readouts of degenerated type II probes do not depend on the concordance between the methylation status of the interrogated dinucleotide and adjacent CpG.

### Substitutions in the second position of the interrogated CpG site

Substitutions in the second position of the interrogated CpG site (G>A, G>T or G>C) have a strong impact on methylation measurements using Infinium probes. They all act in a way similar to SNPs complementary to the 3′-most end of the probe, by affecting the hybridization and thus strongly lowering or completely blocking the efficiency of the probe’s extension. This results in very low fluorescence readouts for any type of the Illumina probes (type I—U and M, and type II), affecting the calculation of β value and returning false estimates of the methylation level (see below). It has to be emphasized that any substitution in the second position of the CpG affects also the first position of the interrogated dinucleotide in the bisulfite-converted DNA; e.g. genomic CpA becomes TpA.

### Summary estimates versus individual beta values and normalized fluorescence redouts

In the routine analysis of the data from Illumina HM450 array, an average methylation level in each studied group (β_av_ or M_av_) is calculated, and used to assess the population/group difference of the methylation status for each probe [13, 22]. This approach is straightforward if the interrogated CpGs are not polymorphic at the level of a genomic sequence. The examples presented above explain some of the mechanisms, by which SNPs in the interrogated dinucleotides may exert an impact on the results of the comparative methylation studies using Illumina Infinium probes. In a population with the high frequency of a CpG allele, the β_av_ value depends on its methylation status. However, in a population with the high frequency of a non-CpG allele, the β_av_ value does not reflect the real (un)methylation status. The analysis of the distribution of individual β values, as presented above, provides important clues for discriminating between β_av_ differences due to the methylation status and to the genomic polymorphisms affecting the analyzed CpG.

A clear tri-modal distribution of individual β values almost invariably indicates the presence of a genomic polymorphism in the first position of CpG sites interrogated by type II probes. The discussed distributions, directly reflecting the distribution of genotypes of the polymorphic positions, can only be observed if CpG alleles are methylated. In this context it has to be remembered that sequences targeted by type II probes, typically located outside of the promoter regions, are more likely to be methylated than sequences examined by type I probes, and thus probably more prone to the discussed effects. A bi-modal distribution often reflects the presence of a SNP in the second position of CpGs interrogated by type I or type II probes, or the presence of a SNP in the first position of CpG interrogated by some of type I probes.

Another alerting distribution is characterized by the increase of individual β values in samples where CpG dinucletide is mutated to a non-CpG allele (and therefore is not “methylable”). In these cases it is advisable to inspect individual values of normalized fluorescence signals in search for cases where the sum of intensities of both fluorescence signals (green and red) are abnormally low compared to other samples. When Infinium probes efficiently hybridize to the interrogated locus, the increase of the green signal indicating methylation occurs at the cost of the red signal indicating lack of methylation, and vice-versa. In case of an inefficient hybridization of the probes (which can be caused by the presence of SNPs but also other confounding factors), individual beta values are artificially inflated, leading to a high β_av_ value estimate, which does not reflect real methylation status of the analysed population/group. It is important to realize that this effect can be only appreciated when individual fluorescence signals are examined.

## Conclusions

### Impact of SNPs in CpGs on comparative population studies

Some observations regarding the impact of SNPs on the results from Illumina HM450K array have been reported in earlier studies on methylation in tissues and phenotypes [12, 16, 19], but this issue was never examined in detail. In our study, which, unlike the previous ones, was based on the inter-population comparisons, the effect of SNPs was more conspicuous due to the high level of SNPs heterozygosity in the examined groups. In the analyzed set of 96 probes, preliminarily selected as differentiating two analyzed populations (Mdiff >1), 66 (over 68 %) interrogated CpGs carrying frequent SNPs with strongly differentiating allele frequencies. It can be envisioned that a similar proportion (although not necessarily the same CpGs) would be affected by SNPs in other inter-population comparisons. Therefore, a conscious interpretation of Illumina HM450K array is an imperative in comparative methylation studies.

The recently published list of Infinium probes affected by factors that confound methylation readouts in Illumina HM450K array [19], is a useful reference. However, one has to keep in mind that, in that study, the selection of probes affected by SNPs in the interrogated CpGs has been based on the 135 release of the Ensembl Database, with some of the SNPs still not validated. Moreover, some populations have a specific distribution of SNP alleles, and in comparative population studies on methylation, the impact of genomic SNPs will depend on the population frequency of the polymorphism. Therefore, while in silico analysis based on the available resources (like the Naeem’s list of Illumina probes) should allow rejection of the majority of common SNPs, some non-recognized SNPs may still affect data analysis. Our study clearly indicates that the observation of a bi-, and especially a tri-modal distribution of β values should prompt a careful inspection of the results from Illumina Bead Chip microarrays. In some cases, genotyping of the analyzed samples should be recommended to check for the presence of a still undiscovered genomic variability. On the other hand, we do not recommend blindly discarding all the probes from the Naeem’s list [19]. In this context it is worth mentioning that cgx* probe mentioned above had been marked on that list to be “discarded”, based on the presence of a SNP in the interrogated CpG. However, the SNP in question had a very low frequency of the non-CpG allele, and in both populations analyzed in our study, the interrogated CpG site was not polymorphic at all, indicating that the observed variation in β values was related to real inter-population differences in the level of methylation.

It has been estimated that only a small fraction of CpGs across the genome vary in their methylation across human populations [13, 22]. In line with these observations, our data demonstrate that all CpG sites without any confounding effects, and thus supposedly indicating true methylation status of the samples, represent very low inter-population differences, with M_dif_ in the range of 1.1–2.9 (manuscript in preparation). Our data suggest that the best candidates for comparative methylation studies could be found among probes with the “cloudy” distribution of individual β values. Some probes with such a pattern were classified as “clean” (free of SNP and other confounding effects); others had SNPs in the interrogated CpGs. The observed cloudy pattern in the latter could be due to overlapping effects, with the methylation differences “masked” by the presence of a SNP. Discarding such probes (solely based on the distribution of B values, with no confirmation of the existence of SNP in the interrogated sequence) may lead to missing some possibly important methylation differences. Inspection of individual probes in search for a “cloudy” appearance of the β values distribution may help to find out, which probes should be retained for the analysis, even if a SNP is present.

Accounting for the presence of SNPs potentially confounding the readout of methylation signals should be mandatory in any research involving comparison of methylation level in populations. This may be crucial not only in comparative population studies, but also in a medical research during control group selection, when a natural SNP variation existing in a population could be incorrectly interpreted as revealing methylation state associated with the disease.

## Methods

### Samples processing

Human B-lymphocyte cell lines representing Caucasian from Georgia (*n* = 18), further referred to as Europeans and Han Chinese from Bejing, further referred to as East Asians (*n* = 18) populations were used in the study. All lines were derived from unrelated healthy adults males (Europeans: 22–56 years old; mean 44 + − 9.4; Chinese: over 18 years old). The cells were cultured under the same standard conditions and harvested at 1 million cell lines per 1 ml. Shortly, immortalized B lymphocytes were purchased from Coriell Cell Repositories. Cells were grown in RPMI medium supplemented with 10 % FBS, 2 % glutamine and 1 % HEPES as well as 1 % penicillin/streptomycin solution (all from Sigma—Aldrich, Steinheim, Germany). Cell lines were maintained in suspension in T flasks in a humidified 5 % CO_2_ atmosphere at 37 °C.

DNA samples isolated from the cell lines were bisulfite converted (according to Zymo Research protocol) and analysed using Illumina Infinium HumanMethylation450 BeadChip array (HM450K, Illumina Inc). Samples were evenly distributed between three slides: on each slide 6 European and 6 Asian samples were analysed. The quality control was performed in Genome Studio Methylation Module (Illumina), according to the Illumina recommendations. All the samples passed the quality criteria.

Genotyping of genomic SNPs in CpG loci was based on the 1000 Genomes database resources and/or direct sequencing of PCR products (primers and PCR conditions are available from the authors upon request).

The study was approved by the Bioethical Committee at the Central Clinical Hospital of the Ministry of Interior in Warsaw (No 67/2100).

### Data analysis

The data were preprocessed using the quantile normalization method SWAN (Subset-quantile Within Array Normalisation) [23], implemented in the minfi Bioconductor package [24]. The widely accepted and used SWAN method was selected because it improves the results obtained from 450 k array and leads to better detection of differential methylations, by reducing the technical variation within and between arrays whilst maintaining the information related to important biological features [23].

To evaluate the batch effect, we performed the unsupervised analysis (multidimensional scaling on 1000 probes with the highest variance; done in minfi package). The main source of variation in the dataset represented the difference between European and East Asian population; no batch effect related to the difference between arrays was observed (data not shown).

To check, whether the pre-processing filtering of probes has the significant impact on the results of our analysis, we calculated the detection P for all the probes. There were 2844 failed probes with a detection p-value greater than 0.01 in at least one sample. We filtered out all the 2844 failed probes from the normalized data and applied the Student’s t-test followed by FDR. We applied the criteria described in the paper (q-value < 0.05 and abs(M_diff) > 1) to select differentially methylated CpGs and obtained 76 such probes (instead of 96). However, the four probes that are described in details in the paper passed the criteria of differentially expressed genes. It shows that adding the filtering step does not have an influence on the paper’s conclusions.

For each site and each individual, β values (representing the ratio of intensities between methylated and unmethylated signals) were calculated using the following formula:

$$ \upbeta = \mathrm{signal}\_\mathrm{Meth}\ /\ \left(\mathrm{signal}\_\mathrm{Meth}+\mathrm{signal}\_\mathrm{Unmeth}\right). $$

β values, ranging from 0 (100 % unmethylated) to 1 (100 % methylated), have a more intuitive biological interpretation and were used here for visualization of signals from individual measurements. M values, which are more statistically valid for differential analysis [25, 26], were calculated using the following formula (with a constraint that β value is in the interval between 0.001 and 0.999):

$$ \mathrm{M} = \log 2\ \left[\upbeta\ /\ \left(1\ \hbox{-}\ \upbeta \right)\right] = \log 2\ \left(\mathrm{signal}\_\mathrm{Meth}\ /\ \mathrm{signal}\_\mathrm{Unmeth}\right). $$

To identify differentially methylated CpGs, the Student’s t-test was performed and absolute values of M_av__diff (the differences between average methylation levels in the two populations) were calculated for each interrogated position. After correction for multiple testing (false discovery rate, FDR) [27, 28], only CpG sites characterized by q < 0.05 were retained. The global methylation in each sample was calculated as the mean beta value of all CpG sites. Student’s t-test was performed to test whether there is a significant difference between global methylation levels in both populations.

### Illumina probes

Illumina HM450K array consists of two assays, Infinium I and II (see also Tables 1 and 2). Infinium I assay involves two types of probes, U and M, complementary to unmethylated and methylated sequence of interest, respectively. The U and M probe sequences, complementary to the interrogated CpG and to the downstream genomic sequence, differ both at their 3′ ends (complementary to the targeted CpG site) and at all positions complementary to other neighboring CpG sites covered by the probe sequence. According to the co-methylation principle [29], methylation status of any neighboring CpG dinucleotide is consistent with that of the interrogated CpG site. Thus, U probes are complementary to the unmethylated CpG sites (converted to TpGs after bisulfite treatment of the genomic DNA) along their whole sequence, whereas M probes are complementary to the methylated CpG sites (not affected by the bisulfite treatment). Methylation status is assessed by comparing fluorescence signals resulting from a single base extension (determined by the adjacent upstream DNA sequence) of both probes (M vs U), after their specific hybridization to, respectively, methylated or unmethylated CpG locus. Infinium II assay involves only one probe per interrogated CpG locus. Probe’s sequence is complementary to the genomic sequence downstream from the targeted CpG (the 3′-most position of a type II probe is complementary to the second position of the interrogated CpG); the probe’s sequence is degenerated (CpG/TpG) at each of the neighboring CpGs. Methylation status is measured based on a single nucleotide extension complementary to the 1st position of the interrogated CpG, depending on the methylation status in the bisulfite-converted DNA; differentially labeled G or A indicate methylated or unmethylated status, respectively. For both types of Infinium probes, the quantitative assessment of signals generated by probes for each of the interrogated CpGs is reported.

Throughout this text, we use the term “under-a-probe” to describe the genomic sequence complementary to that of the probe, but excluding the interrogated CpG.

### Availability of supporting data

The data discussed in this publication have been deposited in NCBI’s Gene Expression Omnibus [30] and are accessible through GEO Series accession number GSE73901 (http://www.ncbi.nlm.nih.gov/geo/query/acc.cgi?acc=GSE73901).

## References

[1] Dempster EL, Pidsley R, Schalkwyk LC, Owens S, Georgiades A, Kane F, Kalidindi S, Picchioni M, Kravariti E, Toulopoulou T, Murray RM, Mill J (2011). Disease-associated epigenetic changes in monozygotic twins discordant for schizophrenia and bipolar disorder. Hum Mol Genet.

[2] Hansen KD, Timp W, Bravo HC, Sabunciyan S, Langmead B, McDonald OG, Wen B, Wu H, Liu Y, Diep D, Briem E, Zhang K, Irizarry RA, Feinberg AP (2011). Increased methylation variation in epigenetic domains across cancer types. Nat Genet.

[3] Meissner A, Gnirke A, Bell GW, Ramsahoye B, Lander ES, Jaenisch R (2005). Reduced representation bisulfite sequencing for comparative high-resolution DNA methylation analysis. Nucleic Acids Res.

[4] Zuo T, Tycko B, Liu TM, Lin JJ, Huang TH (2009). Methods in DNA methylation profiling. Epigenomics.

[5] Brinkman AB, Simmer F, Ma K, Kaan A, Zhu J, Stunnenberg HG (2010). Whole-genome DNA methylation profiling using MethylCap-seq. Methods.

[6] Gupta R, Nagarajan A, Wajapeyee N (2010). Advances in genome-wide DNA methylation analysis. Biotechniques.

[7] Laird PW (2010). Principles and challenges of genomewide DNA methylation analysis. Nat Rev Genet.

[8] Bibikova M, Barnes B, Tsan C, Ho V, Klotzle B, Le JM, Delano D, Zhang L, Schroth GP, Gunderson KL, Fan JB, Shen R (2011). High density DNA methylation array with single CpG site resolution. Genomics.

[9] Sandoval J, Heyn H, Moran S, Serra-Musach J, Pujana MA, Bibikova M, Esteller M (2011). Validation of a DNA methylation microarray for 450,000 CpG sites in the human genome. Epigenetics.

[10] Irizarry RA, Ladd-Acosta C, Wen B, Wu Z, Montano C, Onyango P, Cui H, Gabo K, Rongione M, Webster M, Ji H, Potash JB, Sabunciyan S, Feinberg AP (2009). The human colon cancer methylome shows similar hypo- and hypermethylation at conserved tissue-specific CpG island shores. Nat Genet.

[11] Kraszewska MD, Dawidowska M, Larmonie NS, Kosmalska M, Sędek Ł, Szczepaniak M, Grzeszczak W, Langerak AW, Szczepański T, Witt M (2012). Polish Pediatric Leukemia Lymphoma Study Group: DNA methylation pattern is altered in childhood T-cell acute lymphoblastic leukemia patients as compared with normal thymic subsets: insights into CpG island methylator phenotype in T-ALL. Leukemia.

[12] Chen YA, Lemire M, Choufani S, Butcher DT, Grafodatskaya D, Zanke BW, Gallinger S, Hudson TJ, Weksberg R (2013). Discovery of cross-reactive probes and polymorphic CpGs in the Illumina Infinium HumanMethylation450 microarray. Epigenetics.

[13] Heyn H, Moran S, Hernando-Herraez I, Sayols S, Gomez A, Sandoval J, Monk D, Hata K, Marques-Bonet T, Wang L, Esteller M (2013). DNA methylation contributes to natural human variation. Genome Res.

[14] Majchrzak-Celińska A, Paluszczak J, Kleszcz R, Magiera M, Barciszewska AM, Nowak S, Baer-Dubowska W (2013). Detection of MGMT, RASSF1A, p15INK4B, and p14ARF promoter methylation in circulating tumor-derived DNA of central nervous system cancer patients. J Appl Genet.

[15] Szmida E, Karpiński P, Leszczynski P, Sedziak T, Kielan W, Ostasiewicz P, Sasiadek MM (2015). Aberrant methylation of ERBB pathway genes in sporadic colorectal cancer. J Appl Genet.

[16] Price ME, Cotton AM, Lam LL, Farré P, Emberly E, Brown CJ, Robinson WP, Kobor MS (2013). Additional annotation enhances potential for biologically-relevant analysis of the Illumina Infinium HumanMethylation450 BeadChip array. Epigenetics Chromatin.

[17] Dedeurwaerder S, Defrance M, Calonne E, Denis H, Sotiriou C, Fuks F (2011). Evaluation of the infinium methylation 450 K technology. Epigenomics.

[18] Dedeurwaerder S, Defrance M, Bizet M, Calonne E, Bontempi G, Fuks F (2014). A comprehensive overview of Infinium HumanMethylation450 data processing. Brief Bioinform.

[19] Naeem H, Wong NC, Chatterton Z, Hong MK, Pedersen JS, Corcoran NM, Hovens CM, Macintyre G (2014). Reducing the risk of false discovery enabling identification of biologically significant genome-wide methylation status using the HumanMethylation450 array. BMC Genomics.

[20] Touleimat N, Tost J (2012). Complete pipeline for Infinium(®) Human Methylation 450 K BeadChip data processing using subset quantile normalization for accurate DNA methylation estimation. Epigenomics.

[21] Biswas S, Scheinfeldt LB, Akey JM (2009). Genome-wide Insights into the patterns and determinants of fine-scale population structure in humans. Am J Hum Genet.

[22] Byun HM, Siegmund KD, Pan F, Weisenberger DJ, Kanel G, Laird PW, Yang AS (2009). Epigenetic profiling of somatic tissues from human autopsy specimens identifies tissue- and individual-specific DNA methylation patterns. Hum Mol Genet.

[23] Maksimovic J, Gordon L, Oshlack A (2012). SWAN. Subset-quantile within array normalization for illumina infinium HumanMethylation450 BeadChips. Genome Biol.

[24] Aryee MJ, Jaffe AE, Corrada-Bravo H, Ladd-Acosta C, Feinberg AP, Hansen KD, Irizarry RA (2014). Minfi: a flexible and comprehensive Bioconductor package for the analysis of Infinium DNA methylation microarrays. Bioinformatics.

[25] Du P, Zhang X, Huang CC, Jafari N, Kibbe WA, Hou L, Lin SM (2010). Comparison of Β-value and M-value methods for quantifying methylation levels by microarray analysis. BMC Bioinformatics.

[26] Marabita F, Almgren M, Lindholm ME, Ruhrmann S, Fagerström-Billai F, Jagodic M, Sundberg CJ, Ekström TJ, Teschendorff AE, Tegnér J, Gomez-Cabrero D (2013). An evaluation of analysis pipelines for DNA methylation profiling using the Illumina HumanMethylation450 BeadChip platform. Epigenetics.

[27] Storey JD (2003). The positive false discovery rate: a Bayesian interpretation and the q-value. Ann Stat.

[28] Storey JD, Tibshirani R (2003). Statistical significance for genome-wide studies. Proc Natl Acad Sci U S A.

[29] Eckhardt F, Lewin J, Cortese R, Rakyan VK, Attwood J, Burger M, Burton J, Cox TV, Davies R, Down TA, Haefliger C, Horton R, Howe K, Jackson DK, Kunde J, Koenig C, Liddle J, Niblett D, Otto T, Pettett R, Seemann S, Thompson C, West T, Rogers J, Olek A, Berlin K, Beck S (2006). DNA methylation profiling of human chromosomes 6, 20 and 22. Nat Genet.

[30] Edgar R, Domrachev M, Lash AE (2002). Gene Expression Omnibus: NCBI gene expression and hybridization array data repository. Nucleic Acids Res.

